# Genome-wide SNP analyses reveal high gene flow and signatures of local adaptation among the scalloped spiny lobster (*Panulirus homarus*) along the Omani coastline

**DOI:** 10.1186/s12864-018-5044-8

**Published:** 2018-09-19

**Authors:** Rufaida Dhuhai Al-Breiki, Shannon R. Kjeldsen, Hasifa Afzal, Manal Saif Al Hinai, Kyall R. Zenger, Dean R. Jerry, Mohammed Ali Al-Abri, Madjid Delghandi

**Affiliations:** 1 Centre of Excellence in Marine Biotechnology, Sultan Qaboos University, P.O. Box 50, Al-Khoud, 123 Muscat, Sultanate of Oman; 2 College of Agriculture and Marine Sciences, Department of Marine Sciences and Fisheries, Sultan Qaboos University, P.O. Box 34, Al-Khoud, 123 Muscat, Sultanate of Oman; 3 Centre for Sustainable Tropical Fisheries and Aquaculture and College of Science and Engineering, James Cook University, Townsville, QLD 4810 Australia; 4 College of Agriculture and Marine Sciences, Department of Animal and Veterinary Sciences and Technology, Sultan Qaboos University, P.O. Box 34, Al-Khoud, 123 Muscat, Sultanate of Oman

**Keywords:** Scalloped spiny lobster, Population genetic structure, Management unit, SNP, *F*_ST_ outliers

## Abstract

**Background:**

The scalloped spiny lobster (*Panulirus homarus*) is a popular seafood commodity worldwide and an important export item from Oman. Annual catches in commercial fisheries are in serious decline, which has resulted in calls for the development of an integrated stock management approach. In Oman, the scalloped spiny lobster is currently treated as a single management unit (MU) or stock and there is an absence of information on the genetic population structure of the species that can inform management decisions, particularly at a fine-scale level. This work is the first to identify genome-wide single nucleotide polymorphisms (SNPs) for *P. homarus* using Diversity Arrays Technology sequencing (DArT-seq) and to elucidate any stock structure in the species.

**Results:**

After stringent filtering, 7988 high utility SNPs were discovered and used to assess the genetic diversity, connectivity and structure of *P. homarus* populations from Al Ashkharah, Masirah Island, Duqm, Ras Madrakah, Haitam, Ashuwaymiyah, Mirbat and Dhalkut landing sites. Pairwise *F*_ST_ estimates revealed low differentiation among populations (pairwise *F*_ST_ range = − 0.0008 - 0.0021). Analysis of genetic variation using putatively directional *F*_ST_ outliers (504 SNPs) revealed higher and significant pairwise differentiation (*p* < 0.01) for all locations, with Ashuwaymiyah being the most diverged population (Ashuwaymiyah pairwise *F*_ST_ range = 0.0288–0.0736). Analysis of population structure using Discriminant Analysis of Principal Components (DAPC) revealed a broad admixture among *P. homarus*, however, Ashuwaymiyah stock appeared to be potentially under local adaptive pressures. Fine scale analysis using Netview R provided further support for the general admixture of *P. homarus*.

**Conclusions:**

Findings here suggested that stocks of *P. homarus* along the Omani coastline are admixed. Yet, fishery managers need to treat the lobster stock from Ashuwaymiyah with caution as it might be subject to local adaptive pressures. We emphasize further study with larger number of samples to confirm the genetic status of the Ashuwaymiyah stock. The approach utilised in this study has high transferability in conservation and management of other marine stocks with similar biological and ecological attributes.

**Electronic supplementary material:**

The online version of this article (10.1186/s12864-018-5044-8) contains supplementary material, which is available to authorized users.

## Background

Severe decline of many commercial fish stocks highlights the emerging need for sustainable management plans for regulation and conservation of marine biodiversity. Managing marine stocks sustainably is a dynamic process and requires an in-depth understanding of the stock and its spatial boundaries, along with biological, ecological, evolutionary, economic, social or even political factors that influence the fishery [1, 2]. While traditional fishery management plans rely on morphological and demographic aspects of a population such as growth, size, and mortality rates [3, 4], appropriate management should also consider evolutionary criteria, including conservation of genetic diversity and maintenance of sustainable spawning stock biomass [2]. Recent studies have shown the complexity in the population genetic structure of many marine species [5–7]. Generally, marine organisms possess high genetic diversity and show weak population differentiation due to highly dispersive larval stages and relative absence of barriers to dispersal in the marine environment [8–10]. However, seascape factors (e.g. water currents, seafloor features and bathymetry) and environmental attributes can significantly influence rates of gene flow, connectivity and genetic structure in some species. Further, evolutionary processes like genetic drift and selection [11–13] continuously shape the genomes of marine organisms. For these reasons, defining the population structure of such organisms is challenging, but important for their conservation and management [14–16]. Current progress in the fields of genomics and computational biology doubtlessly offers a versatile platform for fishery managers to answer questions and issues related to population structure, stock boundaries and the level of divergence of marine organisms [1, 16, 17]. Recent reports support the successful application of genomic approaches to identify conservation or management units (MUs) of marine species [8, 18, 19]. Many of these utilise advanced genomic approaches, using high-throughput genotyping technologies i.e. Next generation sequencing (e.g. Illumina HiSeq and MiSeq platforms) and third generation sequencing (e.g. PacBio and Nanopore technology), to isolate large number of genetic markers suitable for inference of population differentiation and structure [20–23]. These technologies have enabled the development of panels of SNP markers to investigate interspecific hybrids [24, 25], to assign individuals to populations, or to identify MUs [8, 11, 26]. Harnessing of genomic wide SNPs in the assessment of commercial marine stocks is a successful approach that could address many questions related to the level of genetic diversity and stability of these stocks [27].

The scalloped spiny lobster *P. homarus* (Linnaeus 1758) is characterised by a relatively long pelagic larval duration (PLD) of about 4.5–6.5 months [28], during which the larvae is exposed to oceanic dispersal as a result of currents and wind-shear, before metamorphosing into the puerulus stage and continuing its life as a benthic organism [28, 29]. The species is distributed throughout the Indo-Pacific [30] and in the region supports valuable fisheries of considerable socio-economic importance. There are major concerns about the future of spiny lobster fisheries owing to a general decline in catch [31, 32], emphasizing the need for serious efforts towards sustainable fishery management and regulation of the species. It is essential to introduce comprehensive fishery management guidelines for the species, considering a wide range of biological aspects e.g. demographic interactions of individuals and genetic structuring. Characterisation of stock boundaries and identification of population divergence will greatly support managers in deciding whether two populations should be managed together, or as separate stocks [27, 33]. Many recent works of *P. homarus* have studied sub-species resolution, phylogeography throughout its wide range [34–37] or dispersal capabilities [38]. However, the fine-scale genetic structure of the species in many regions is still remains unrevealed [38].

Commercial spiny lobster fisheries have a long tradition in Oman, with the country currently being one of the major suppliers to the global market [39]. Of concern, however, is the observation that the annual harvest of Omani lobsters has declined dramatically from 2000 tons/year in the 1980’s, to less than 485 tons in 2016 [40]. Presently, the lobster fishery management in Oman is primarily based on data from growth, mortality and catch rates and aims to increase population densities [39, 41]. Despite regulations implemented by the government, they are not regularly reinforced (i.e. high incidence of illegal catch) and no clear legislation system against illegal practices [42]. There is a lack of knowledge surrounding the population genetic structure of *P. homarus* in Oman, its levels of genetic fitness and relatedness in this region. Hence, the lobster stock along the Omani coastline is currently treated as one single management unit. This study is the first to assess the genetic structure of *P. homarus* in Oman using high resolution genome-wide SNPs genotyping. The findings provide valuable insight into the connectivity of the Omani *P. homarus* population and will aid in the identification of management units for the fishery of this commercially important crustacean.

## Results

### SNPs quality control and filtering

A primary dataset of 48,140 SNPs was filtered to retain 7988 SNPs (Additional file 1) suitable for genomic analysis (Table 1). Significant deviations from Hardy Weinberg Equilibrium (HWE) were observed across all populations (*p* < 0.000004 after Bonferroni correction). After the removal of these SNP loci deviating from HWE, significant skew in estimation of genetic diversity indices (*F*_is_ and *H*_o_) was observed. Thus, indicating the status of those SNPs as putative null alleles or genotypic artifacts. Additionally, nine individuals were excluded from the dataset due to poor genotyping coverage < 80%.

**Table 1:** Filtering steps and SNPs counts retained after each step

	Retained SNPs count
Initial potential SNPs	48,140
Duplicated SNPs filters	39,086
Clustered SNPs filters	32,840
Call rate ≥ 0.7	23,764
Replication average ≥ 0.95	23,549
SNPs coverage ≥80%	20,421
Reads depth ≥ 5	14,695
SNPs MAF ≥ 0.02	12,589
HWE filters	7988
Retained SNPS for genomic analysis	7988

*MAF* minor allele frequencies, *HWE* Hardy Weinberg Equilibrium

### Population genetic diversity

Observed heterozygosity (H_o_) across populations ranged from 0.1660 to 0.1840 and were generally lower than the expected heterozygosity values (*H*_n.b_) (0.2260–0.2333, Table 2). Average individual multilocus heterozygosity (Av.MLH) revealed similar values and distribution to H_o,_ ranging from 0.1683 to 0.1858 (for Ashuwaymiyah and Haitam, respectively) (Table 2). Average standardized MLH (sMLH) values slightly varied across populations and ranged from 0.9302 to 1.029. Inbreeding coefficient (*F*_is_) was significantly high across all populations, (0.2094–0.2861, Table 2). The estimated parameters of identity disequilibrium (g^2^) slightly differentiated from zero (0.0003–0.0038). However, this differentiation was statistically significant (i.e. 95% C. I. does not overlap zero) in two locations (Haitam and Dhalkut, Table 2). Estimated effective population size (N_*eLD*_) varied from 5507.5 for Haitam and 10,305.8 for Al Ashkharah to an infinite value for other populations.

**Table 2:** Genetic diversity indices for *P. homarus* for each sampling site using 7988 SNPs

Location	n	*H*_n.b_ (±SD)	*H*_o_ (±SD)	Av. MLH (±SD)	sMLH (±SD)	g^2^ (95% C.I.)	*F*_is_ (*p* < 0.001)	*N*_*eLD*_ (95% C.I.)
Al Ashkharah	20	0.2324 (±0.1643)	0.1789 (±0.1495)	0.1807 (±0.0027)	1.001 (±0.0147)	0.0038 (0.0000–0.0088)	0.2349	10,305 (4860 - ∞)
Masirah	29	0.2277 (±0.1635)	0.1803 (±0.1481)	0.1823 (±0.0009)	1.009 (±0.0053)	0.0004 (0.0000–0.0008)	0.2113	∞ (∞ - ∞)
Duqm	19	0.2275 (±0.1691)	0.1798 (±0.1532)	0.1817 (±0.0011)	1.005 (±0.0062)	0.0003 (−0.0001–0.0008)	0.2142	∞ (∞ - ∞)
Ras Madrakah	17	0.2260 (±0.1715)	0.1799 (±0.1576)	0.1820 (±0.0013)	1.006 (±0.0073)	0.0006 (0.0000–0.0013)	0.2094	∞ (∞ - ∞)
Haitam	20	0.2333 (±0.1630)	0.1840 (±0.1509)	0.1858 (±0.0015)	1.029 (±0.0084)	0.0010 (0.0005–0.00151)	0.2160	5507 (3457–13,521)
Ashuwaymiyah	10	0.2287 (±0.1843)	0.1660 (±0.1677)	0.1683 (±0.0025)	0.9302 (±0.0144)	0.0031 (−0.0002–0.0077)	0.2861	∞ (∞ - ∞)
Mirbat	20	0.2270 (±0.1678)	0.1780 (±0.1513)	0.1797 (±0.0024)	0.9947 (±0.0131)	0.0044 (−4.8190–0.0122)	0.2203	∞ (∞ - ∞)
Dhalkut	29	0.2273 (±0.1640)	0.1770 (±0.1437	0.1788 (±0.0017)	0.9892 (±0.0094)	0.0032 (0.0007–0.0059)	0.2248	∞ (∞ - ∞)

*n* number of samples, *H*_n.b_ average expected heterozygosity corrected for population sample size, *H*_o_ observed heterozygosity, Av. MLH average multi-locus heterozygosity, sMLH standard multi-locus heterozygosity, g^2^ identity disequilibrium parameter, *F*_is_ inbreeding coefficient, *N*_*eLD*_ effective population size by the linkage disequilibrium method with 95% confidence interval

### Population differentiation and genetic structure

**Fig. 1:**
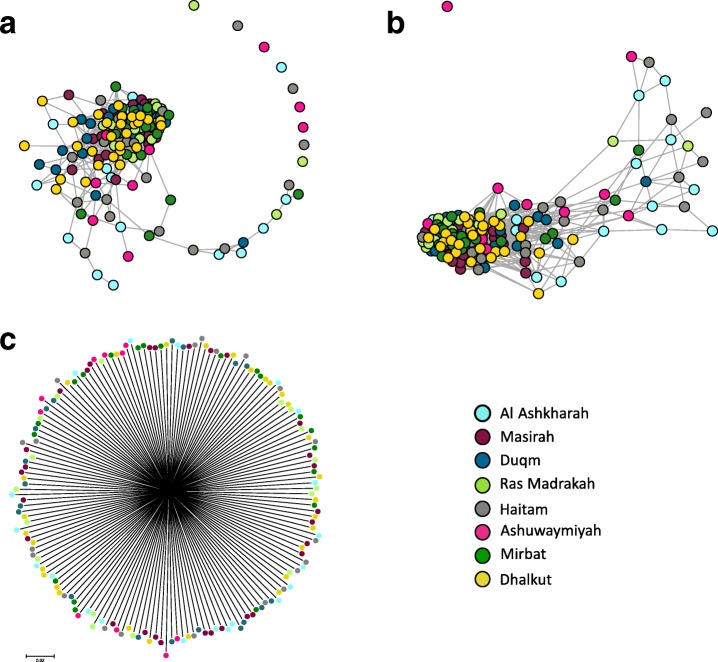
Population structure of 164 individuals of *P. homarus* samples using 7988 SNPs. Population network was constructed using NetView R v.1.0. The network is visualised at nearest neighbor (k-NN) threshold of 25 (**a**) and 50 (**b**). Un-rooted neighbor-joining tree (**c**) was drawn in MEGA6 using 1-psa genetic distances, scale bar indicates genetic distance

In general, pairwise genetic differentiation estimates (*F*_ST_) using 7988 SNPs indicated very low levels of genetic differentiation, with average *F*_ST_ = 0.0004 (±SD = 0.1843), with only seven out of 28 pairwise comparisons being statistically significant (Table 3). AMOVA indicated an absence of hierarchical genetic structure between populations (variation of 80.47% within individuals; 19.51% among individuals; 0.01% among populations and 0% among groups (AS, MA; group1; DU, RM, group2; HA, SH, group3; MI, DA; group4)). While visualization of population structure using DAPC with all 7988 SNPs revealed two admixed genetic clusters (Additional file 2), population Network analysis with Netview R displayed only one genetic cluster (Fig. 1a, b). Similarly, high admixture was observed through the NJ tree, indicating high genetic relatedness among individuals from different geographical locations (similar branch lengths among all individuals, Fig. 1c).

**Table 3:** Pairwise *F*_ST_ values for 7988 SNPs using Genetix v.4.05.2 with permuted *p*-values inside brackets

	Al Ashkharah	Masirah	Duqm	Ras Madrakah	Haitam	Ashuwaymiyah	Mirbat
Masirah	−0.0006 (0.970)	–	–	–	–	–	–
Duqm	0.0001 (0.570)	0.0007 (0.198)	–	–	–	–	–
Ras Madrakah	0.0004 (0.416)	−0.0002 (0.347)	0.0007 (0.306)	–	–	–	–
Haitam	0.0003 (0.319)	0.0002 (0.477)	0.0013^a^ (0.044)	0.0001 (0.690)	–	–	–
Ashuwaymiyah	0.0006 (0.267)	0.0002^a^ (0.045)	0.0016^a^ (0.041)	0.0021^a^ (0.010)	0.0021^a^ (0.031)	–	–
Mirbat	−0.0008 (0.931)	−0.0004 (0.901)	0.0013^a^ (0.040)	−0.0001 (0.771)	− 0.0001 (0.720)	0.0004 (0.258)	–
Dhalkut	−0.0003 (0.485)	0.0005 (0.129)	0.0005 (0.198)	−0.0001 (0.780)	0.0007 (0.112)	0.0021^a^ (0.043)	0.0007 (0.491)

^a^denotes significant comparisons

### Putatively selective SNPs

**Fig. 2:**
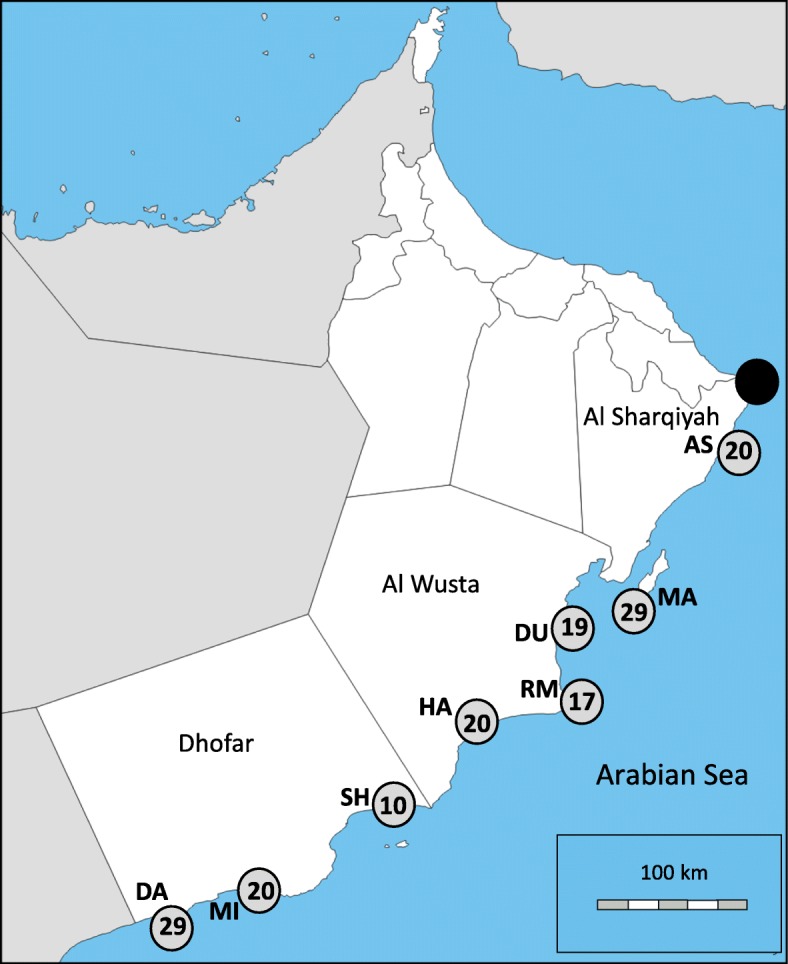
Sampling sites of *P. homarus* specimens along the coastline of Oman. **AS** Al Ashkharah, **MA** Masirah Island, **DU** Duqm, **RM** Ras Madrakah, **HA** Haitam, **SH** Ashuwaymiyah, **MI** Mirbat and **DA** Dhalkut. Numbers in circles represent genotyped samples in the final analyses. The black circle represents Ras Al-Had, the northern border of the commercial fishery sites of *P. homarus*. The map was obtained from: (https://www.d-maps.com/carte.php?num_car=516&lang=en) and edited to highlight the sampling sites

**Fig. 3:**
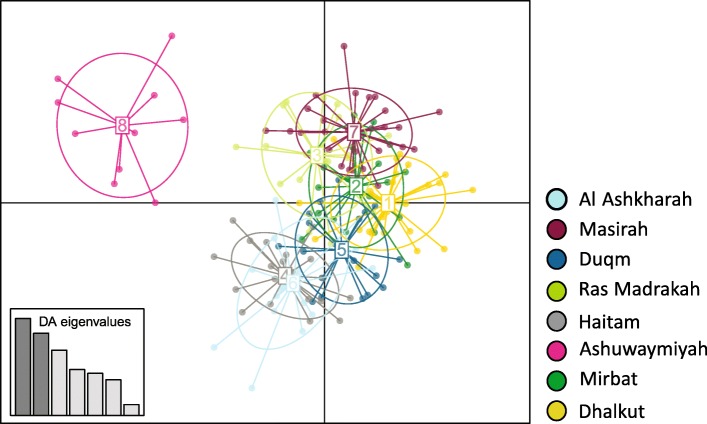
DAPC scatter plot created using eight optimum PCs out of 504 outlier SNPs across 164 *P. homarus* individuals in the R package *adegenet*. Dots represent individuals. The plot showing Ashuwaymiyah represents a distinct genetic cluster and other sampling locations represent a second admixed genetic cluster

**Fig. 4:**
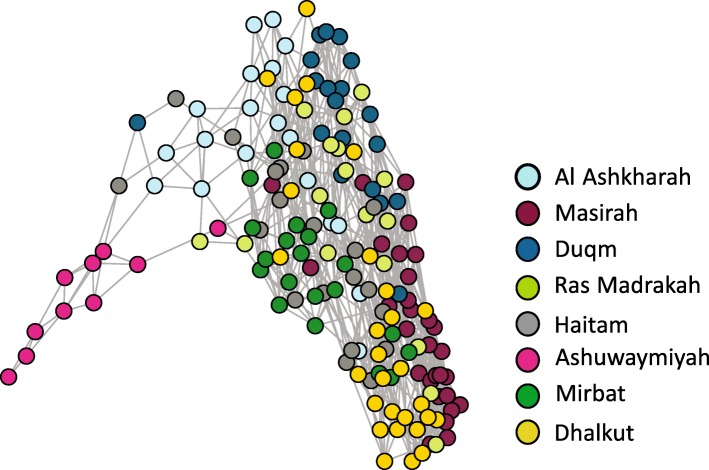
Population network of *P. homarus* individuals using NetView R v.1.0 based on identity by similarity (IBS) distance matrix calculated in PLINK after Steinig et al. [104]. The network was drawn at nearest neighbor (k-NN) threshold of 15, using 504 potentially directional SNPs and 164 individuals

The genomic scan using Bayescan v.2.1 identified only one SNP as a directional outlier, which was characterized by Jeffery’s scale as decisive. The frequentist approach with Arlequin v.3.5.2.2 indicated out of 7988 SNPs, 504 SNPs (6.31%) potentially under divergent selection, 168 SNPs (2.10%) under balancing selection and 7316 SNPs (91.59%) likely to be neutral (Genotypes of 504 SNP loci are available as Additional file 3). Global *F*_ST_ for the 504 SNPs candidate outliers was 0.0423 (Table 4), more than 100 times greater than for all 7988 SNPs (0.0004). All of the pairwise estimates with these directional outliers were significant (*p* < 0.001), with highest differentiation between Ashuwaymiyah and Ras Madrakah (0.0736) and the lowest between Masirah and Dhalkut (0.0288) (Fig. 2; Table 4). Visualization of DAPC revealed that Ashuwaymiyah samples represented a distinct genetic cluster, while all other samples were grouped into a second admixed genetic cluster (Fig. 3). The same pattern was also observed by population Netview at a range from k-NN =10 to 30 and visualized at k-NN = 15 (Fig. 4).

**Table 4:** Pairwise *F*_ST_ values for 504 directional SNPs using Genetix v.4.05.2

	Al Ashkharah	Masirah	Duqm	Ras Madrakah	Haitam	Ashuwaymiyah	Mirbat
Masirah	0.0366	–	–	–	–	–	–
Duqm	0.0407	0.0457	–	–	–	–	–
Ras Madrakah	0.0423	0.0678	0.0489	–	–	–	–
Haitam	0.0325	0.0406	0.0446	0.0411	–	–	–
Ashuwaymiyah	0.0662	0.0678	0.0696	0.0736	0.0664	–	–
Mirbat	0.0383	0.0322	0.0476	0.0437	0.0343	0.0706	–
Dhalkut	0.0352	0.0288	0.0397	0.0387	0.0332	0.0668	0.0309

All pairwise *F*_ST_ values are significant (actual permuted *p-*values < 0.001 for all estimates)

## Discussion

### Population genetic diversity

Assessment of genetic diversity and population structure of *P. homarus* along the Omani coastline is vital for its stock management and conservation. This study is the first to identify a genome-wide SNP dataset for *P. homarus* utilising DArTseq technology and to determine the genetic stock structure of the species in Oman. The obtained SNP dataset revealed unique insights into genetic diversity and stocks boundaries of the spiny lobster in Oman. Significant deviations from Hardy Weinberg Equilibrium (HWE) were observed across all populations, apparently caused by a heterozygote deficit as represented by significantly high *F*_is_ values (all *F*_is_ values > 0, *p* < 0.001). Despite repetitive culling of SNPs deviating from HWE, notably lower, but still significantly high *F*_is_ values (0.2094–0.2861) were observed. Heterozygote deficits have been observed in many marine invertebrates [43] and are believed to be caused by genotypic artifacts, null alleles, population stratification caused through the Wahlund effect, biological and behavioural traits and selection [44–46]. In other cases, genotype calling errors have been reported to cause deviations from HWE [47–49]. To overcome these in our dataset, SNP loci which had call rate (< 0.7) and those significantly deviated from HWE or had low minor allele frequencies (MAF) were removed [48]. In this study, estimations of identity disequilibrium (g^2^) slightly differentiated from zero (0.0003–0.0038, Table 2) with no significance except for Haitam and Dhalkut, thus, the obtained high *F*_is_ values for *P. homarus* samples in this study are less likely due to inbreeding.

Additionally, average multi locus heterozygosity estimates (Av.MLH) showed a moderate to high level of genomic heterozygosity (0.1683–0.1858) compared to some marine invertebrates e.g. *Hapalochlaena maculosa* (0.0800–0.1720) [50], *Pinctada margaritifera* (0.0520–0.1030) [11] and *P. maxima* (0.3030–0.3110) [51]. This may support that the significantly high *F*_is_ values are unlikely due to reduction in genomic heterozygosity. Multi locus heterozygosity may reflect genome wide heterozygosity especially when thousands of genomic markers are used [52, 53].

It is also unlikely that the high *F*_is_ values are caused by the Wahlund effect, as Netview analyses indicated high levels of admixture (Fig. 1a, b). Possibly, the observed heterozygote deficits are caused by null alleles or selection. In our study, high missingness rate (> 0.2) of SNPs was observed for some samples from Ashuwaymiyah and were excluded, as they did not match our quality criteria. In the remaining samples (with genotyping coverage ≥0.8), monomorphic loci were observed for Ashuwaymiyah only, but not other sites. The monomorphic loci observed in Ashuwaymiyah samples could be attributed to mutations in restriction enzyme sites during DArTseq genotyping causing null alleles in populations [54]. It is also possible, that these observations were due to small sample size effect from Ashuwaymiyah. A study in our group with microsatellite markers revealed divergence of Ashuawymiyah stock in a larger sample size of 20 individuals (Delghandi et al., under prep).

Generally, null alleles may indicate occurrence of genetic variation in the form of point mutations or structural mutations (i.e. insertions/deletions) and contribute to the organism’s fitness and adaptation [55]. It has been reported that the frequency of missing data is correlated with the level of genetic divergence between populations [54, 56]. Other studies, however, have reported that null alleles are frequently encountered in SNP datasets and generate biases in estimation of diversity indices [57].

### Population differentiation and genetic structure

In our study, pairwise differentiation estimates (*F*_ST_) analysis using 7988 SNP loci, showed low but statistically significant (*p* < 0.05) differentiation between Ashuwaymiyah and other locations, except for Al Ashkharah and Mirbat (Table 3). Earlier reports showed that *P. homarus* possesses a genetic structure at the large Indo-Pacific scale [36] and relatively small scales, within both the north west Indian Ocean and south west Indian Ocean [38, 58].

Other studies demonstrated different patterns and levels of genetic structure in spiny lobsters e.g. *P. ornatus*, exhibit low levels of genetic structure across an expansive distribution of the Indo-Pacific [59], but not at smaller scales i.e. South East Asia [60]. Similarly, no genetic structure was observed in Hawaiian *P. penicillatus* [61], while at the scale of the Indian Ocean and across its Indo-Pacific range, significant structure was revealed [62, 63]. These contrasting patterns are referred to, mainly, environmental (e.g. pattern of water circulation) [38], bioecological (e.g. larval retention) [64], behavioral (e.g. spawning migrations) [60], or geographical factors (e.g. habitats patchiness) [59]. In this study, the observed genetic divergence among Omani *P. homarus* could be caused by distinct environmental and geographical factors in the region. The Omani coast in the Arabian sea is known to be influenced by complex water circulation which varies seasonally with the Monsoon and results in a series of eddies along the coast of Oman [65, 66]. Generally, eddies might act as a larval retention system [67], limiting larval dispersal and maintaining divergence in marine populations including spiny lobsters, e.g. *Jasus edwardsii* [68] and *P. h. rubellus* [38]. Additionally, fragmentation of marine habitats across the Omani coastline by sandy stretches and absence of corals and rock reefs [44] could have contributed to this observation. Similar observations have been reported for other marine organisms with larval life stage such as Corkwing wrasse across Norwegian coastline [69] and the Omani clownfish [70]. In fact, Ashuwaymiyah is a shallow bay characterized by large rocky reefs, and its extended sea shelf (about 50 km) is less affected by the Monsoon currents, even during its extremes from June to September (National Survey Authority, Ministry of Defense, Oman, unpublished local data). This unique geography of Ashuwaymiyah could have limited the gene flow among lobsters from Ashuwaymiyah and other sites. This could be a possible explanation for the putative genetic divergence of *P. homarus* samples from Ashuwaymiyah. Moreover, it is not surprising to observe a genetically heterogeneous stock among other admixed stocks over a relatively small geographical scale. Such observation of fine scale genetic differentiation within relatively high gene flow environment has been widely described in a variety of marine species with planktonic early life stage and in a phenomenon known as chaotic genetic patchiness [71, 72]. Examples include crown-of-thorns starfish, *Acanthaster planci* [73], clam, *Spisula ovalis,* [74], the sea urchin, *Strongylocentrotus purpuratus* [75], marine goby, *Coryphopterus personatus* [76], marine goby, pulmonate limpets, *Siphonaria sp*. [71], bicolour damselfish, *Stegastes partitus* [77] and spiny lobster, *P. interruptus* [78]. Therefore, it is possible that the observed slight divergence of Ashuwaymiyah is due to chaotic genetic patchiness. A typical feature of chaotic genetic patchiness is being temporal therefore, repetitive sampling from Ashuwaymiyah would be useful to clarify the current status of the genetic heterogeneity in Ashuwaymiyah.

### Detecting potential selective SNPs

Genome-wide scan for *F*_ST_ outliers using Bayescan v2.1 identified only one directional outlier, hence further analysis of population structure was not possible using this approach. In contrast, applying Arlequin resulted in more candidate outlier loci being identified, allowing further investigation for adaptive variation. It is common to detect less candidate outliers with Bayescan as it is a conservative approach and may fail to detect relatively low signals of selection [79–81]**.** Therefore other studies have used only the frequentist approach to perform *F*_ST_ outliers analysis [82].

Assessment of population structure with putatively directional SNPs using both population Network and DAPC, revealed Ashuwaymiyah to be potentially under local adaptive pressures (Figs. 3 and 4). The utilization of the whole dataset of SNPs could not capture the low levels of genetic structure, while analyses based on *F*_ST_ outliers allowed detection of selective divergence and identification of possible discrete stock. Similarly, many other studies showed that the use of *F*_ST_ outliers could detect adaptive variation in the absence of broader analyses based on neutral markers [83–85]. A possible explanation for the observed divergence is the heterogeneous environmental attributes i.e. massive rocky reefs and shallow waters in Ashuwaymiyah, which might be the driver of this differentiation. In addition to the morphological/biological differences among *P. homarus* stocks [42], genetic studies with microsatellite markers revealed a significant divergence of lobsters from the Dhofar governorate, including Ashuwamiyah from Al Sharqiyah and Al Wusta governorates (Delghandi et al., under prep).

### Implications for fishery management

The population of *P. homarus* along Oman is currently considered as a single homogenous stock with a single management regulation. This study shows that the samples from Ashuwaymiyah are genetically distinct from other broadly admixed samples, albeit at low levels. An earlier study reported that stock of *P. homarus* in Ashuwaymiyah was significantly differentiated in body size and that lobsters reach maturity at significantly lower sizes when compared to two geographically close locations [86]. The same study suggested a need to investigate the current fishery management further and indicated that Ashuwaymiyah site might require separate management. Additionally, a recent biological study of *P. homarus* in Oman revealed that the stock in Dhofar governorate differs from other stocks in size, time of spawning and number of spawning peaks/year, suggesting a need to consider spatial management of *P. homarus* along the Omani coastline [42]. Our study delivers for the first time genetic support for possible differentiation of the Ashuwamiyah stock from other locations across the coastline of Oman, using genome-wide markers, and that this stock might need to be considered for regional management. We recommend the conduct of further studies with larger number of samples, coupled with environmental and ecological data, to aid integrated assessment studies and potential discovery of unique management units.

## Conclusions

Utilisation of genome wide SNPs to study the genetic status of *P. homarus* stocks in Oman provided valuable insights into the genetic status of the stock. This genomic resource is the first of its kind in *P. homarus* and the SNP dataset obtained in this study has allowed deep characterization of the lobster population genetic diversity, connectivity and structure in Oman. This study has revealed general admixture and high connectivity of *P. homarus* across the Omani coastline. Additionally, the study highlighted the potential prevalence of local adaptive pressures in Ashuwaymiyah. These findings indicate the importance of considering spatially customized management strategies for *P. homarus* across the coastline of Oman. Further studies of the genetic status of Ashuwaymiyah and stocks from other locations in Dhofar with adequate sampling based on different temporal periods together with ecological and environmental data about Omani coastline is required before any conclusive decision on the stock structure can be inferred.

## Methods

### Sampling and genomic DNA extraction

The commercial *P. homarus* fishery in Oman is situated between Ras Al-Hadd and Dhalkut (a distance of approximately 1100 km) (Fig. 2). Samples were obtained from eight commercial landing-sites, covering most of the distribution range of the lobster in Oman (Fig. 2). All samples were euthanized and purchased from local fishermen in March 2015 during the legal fishing season. A single walking leg was excised from wild caught *P. homarus* (*n* = 172) and preserved immediately in 95% ethanol until DNA extraction. All samples were purchased from local fishermen in March 2015 during the legal fishing season. Genomic DNA was extracted from tissue samples using a modified cetyl trimethyl ammonium bromide (CTAB)/ Chloroform-Isoamyl method [87]. DNA extracts were further purified through a Sephadex G50 (GE, 2007) column prior to quantification with a Nanodrop 1000 Spectrophotometer (Thermo Scientific).

### Library preparation and genotyping

Genomic DNA extracts were standardised to 50 ng/μl, and sent for sequencing and genotyping using DArTseq™ technology, with Diversity Arrays Technology, Canberra, Australia [88, 89]. Library preparation was completed as described by Kilian et al. [89] and Sansaloni et al. [90] with all *P. homarus* DNA samples being digested using a combination of *PtsI* and *HpaII* restriction enzymes. Multiplexed reduced representation libraries were then sequenced on the Illumina HiSeq2500 platform for 77 cycles.

To call SNPs and genotype each individual, raw Illumina HiSeq2500 data was first de-multiplexed into individual samples, based on sample-specific barcode sequences. De-multiplexed samples were then assessed for overall sequence quality, with any fragments with an average Q-score of < 25 being removed from the dataset. Sequences were also compared to public databases for identification of contaminant sequences, and any non-target sequences (including bacterial and viral fragments) were removed. SNP calling was conducted using the *DArTsoft14* algorithm within the KDCompute framework developed by Diversity Arrays Technology (http://www.kddart.org/kdcompute.html), with initial calling parameters and filtering methods as described in Morse et al. [50] and Lind et al. [91].

### SNPs quality control and filtering

To eliminate potentially aberrant SNPs, stringent quality controls were applied using custom python scripts within the DArTQC pipeline (https://github.com/esteinig/dartQC) [92]. Initially, all duplicated sequences with > 95% similarity were identified using CD-HIT and collapsed into a single cluster, or removed [93]. Further, SNPs with a call rate < 70% and those where technical replicates did not return a repeatability value of > 95% were also removed. Additionally, individuals and SNPs with > 20% missing data and SNPs with a Minor Allele Frequency (MAF) < 0.02 were excluded using Plink v1.07 [94].

To investigate the effect of sequencing depth, *F*_is_ and *H*_o_ were calculated for each population at different reads depth (Average SNP Counts) thresholds (3, 5, 7 and 10) to discover the degree of potential bias caused by lower call depths. Accordingly, four subsets of SNPs were generated at these sequencing depths. To detect potential genotyping artifacts, SNPs were tested for significant deviation from Hardy-Weinberg equilibrium (HWE) using Arlequin v.3.5.2.2 [95]. Any SNP loci which significantly deviated from HWE were excluded following Bonferroni correction (*p* < 0.000004). To assess the impact of deviation from HWE, *F*_is_ and *H*_o_ were calculated before and after removal of significantly deviated SNPs.

### Population genetic diversity

To estimate the genetic diversity within populations, standard allelic diversity indices including average observed heterozygosity (*H*_o_), average expected heterozygosity corrected for population sample size (*H*_n.b._) and inbreeding coefficient (*F*_is_) were calculated using Genetix v.4.05.2 [96]. Effective population size, using a linkage disequilibrium method (*N*_*eLD*_) was computed with NeEstimator [97]. To examine individual genome wide diversity and individual inbreeding, multi-locus heterozygosity (MLH) and identity disequilibrium parameter (g^2^) were calculated for all individuals using the R package inbreedR [52].

### Population differentiation and genetic structure

To assess population differentiation and genetic structure, a number of different statistical approaches were conducted. The extent of pairwise population differentiation was evaluated using Weir and Cockerham’s unbiased F-statistics [98] through Genetix v.4.05.2 [96]. To assess hierarchical levels of population structuring, an analysis of molecular variance (AMOVA) using Arlequin v.3.5.2.2 [99] was calculated between sampling locations based on grouping samples in four groups (AS, MA; group1; DU,RM, group2; HA, SH, group3; MI, DA; group4). The grouping criterion was based on habitat similarity i.e. abundance of corals and rock substrates. Obviously, Al Wusta coast is dominated by sandy habitat and sparse coral colonies [100, 101]. Region surrounding DU has been exposed for the last seven years to major construction and industrial influence, most probably having an impact on fragmentation of marine populations. Hence, HA being not affected of these factors, was grouped with SH, which is just below the border line between Al Wusta and Dhofar governates (Fig. 2). In addition, the function find.clusters in the R package *adegenet* [102] was used to determine the optimal number of clusters with the Bayesian Information Criterion (BIC) method. To assess levels of differentiation between the obtained genetic clusters, Discriminant Analysis of Principal Components (DAPC) was used. DAPC was performed using the optimum number of principal components (PCs) calculated using the α-score function in *adegenet* [103].

Finally, a network analysis with no prior population assumptions was performed to assess both broad and fine scale population structure using NetView R [104]. NetView was run through the R implementation of NetView P [104, 105] at a k-NN range from 25 to 50 as determined by a k-NN selection plot. Similarly, to visualise the extent of relatedness between individuals within each population and divergence among populations, a Neighbour-Joining (NJ) tree was constructed in MEGA6 [106]. The NJ tree was constructed using 1-proportion of shared alleles (1-psa) genetic distance matrix calculated in the R package *adegenet* using propShared function [102].

### Identifying potential selective SNPs

To detect possible signatures of directional and balancing selection, detection of putatively selective outlier SNPs was performed using an *F*_ST_ approach. To minimize false positive rates in identifying SNPs under selection, two independent statistical approaches were used. A Bayesian approach was implemented in Bayescan v.2.1 [107] and a frequentist approach [108] was implemented in Arlequin v.3.5.2.2 [99]. Bayescan estimates population-specific *F*_ST_ coefficients by the Bayesian method described in [109] and uses a statistical cut-off based on the mode of the posterior distribution to detect SNPs under selection [107]. Bayescan v2.1 was used with 1:10 prior odds for a neutral model and all other parameters were kept as default (20 pilot runs of 5000 iterations followed by 100,000 iterations with an additional burn-in of 50,000) [110]. Once probabilities had been calculated for each locus, they were ranked from largest to smallest. SNPs with posterior probabilities ≥0.91–1, which are categorised as strong to decisive according to the Jeffery’s scale [110], were retained. In addition, the Bayescan v2.1 function, plot R.r in the R v.3.3.1 was used to control the false discovery rate (FDR) of the selective markers at FDR of 0.05. SNPs were considered as outliers if their probability was > 0.9 at FDR of 0.05.

The frequentist approach in Arlequin v.3.5.2.2 was executed under a finite island model with 200,000 simulations and 100 demes simulated [99]. SNPs were considered as outliers based on their *F*_ST_ and *p* values. SNPs were considered as directional loci if their *F*_ST_ values fell within the upper 5% quantile and *p* < 0.05. They were considered as balancing SNPs if their *F*_ST_ values fell in the lower 5% quantile and *p* < 0.05.

To assess the population structure based on directional outliers, a dataset of the putative outlier SNPs was generated. Broad scale population differentiation based on this SNP dataset was examined by calculating magnitude and significance of pairwise *F*_ST_ comparisons using Genetix v.4.05.2. Population structure and network were examined based on the putative outlier SNPs using DAPC and Netview respectively. DAPC was visualised after retaining the optimum number of PCs and NetView was run at k-NN range = 10 to 30.

## Additional files

Additional file 1: Genotypic data of *P. homarus* for 164 individuals from Oman using 7988 SNPs. Genotypes are in genetix format. (TXT 576 kb)

Additional file 2: A plot of the Discriminant Analysis of Principal Components (DAPC) against the discriminant function retained. The plot is indicating presence of two genetic clusters of *P. homarus* in Oman. The plot was generated using the most informative 13 PCs identified from all 7988 SNPs dataset across 164 *P. homarus* individuals in the R package *adegenet. (TXT 9105 kb)*

Additional file 3: Genotypic data of *P. homarus* for 164 individuals from Oman using 504 putatively directional SNPs. Genotypes are in genetix format. (PDF 389 kb)

## Abbreviations

1-psa: 1-proportion of shared alleles
AMOVA: Analysis of molecular variance
AS: Al Ashkharah
BIC: Bayesian information criterion
CTAB: Cetyl trimethyl ammonium bromide
DA: Dhalkut
DAPC: Discriminant analysis of principal components
DartQC: Diversity arrays technology SNPs quality control
DArT-seq: Diversity arrays technology sequencing
Du: Duqm
FDR: False discovery rate
gDNA: Genomic deoxyribonucleic acid
HA: Haitam
HWE: Hardy-Weinberg equilibrium
IBS: Identity by similarity
k-NN: Number of nearest neighbours k-threshold
Ma: Masirah Island
MAF: Minor allele frequency
MI: Mirbat
MLH: Multi-locus heterozygosity
NJ: Neighbour-joining
PCR: Polymerase chain reaction
PCs: Principal components
PLD: Pelagic larval duration
RM: Ras Madrakah
SH: Ashuwaymiyah
sMLH: Standardized multi-locus heterozygosity
SNP: Single nucleotide polymorphism

## References

[1] Reiss H, Hoarau G (2009). Ckey-Collas M, Wolff WJ. Genetic population structure of marine fish: mismatch between biological and fisheries management units. Fish Fish.

[2] Lal MM, Southgate PC, Jerry DR, Bosserelle C, Zenger KR (2016). A parallel population genomic and hydrodynamic approach to fishery Management of Highly-Dispersive Marine Invertebrates: the case of the Fijian black-lip pearl oyster Pinctada margaritifera. PLoS One.

[3] Carvalho GR, Hauser L (1994). Molecular-genetics and the stock concept in fisheries. Rev Fish Biol Fish.

[4] McKeown NJ, Arkhipkin AI, Shaw PW (2017). Regional genetic population structure and fine scale genetic cohesion in the southern blue whiting Micromesistius australis. Fish Res.

[5] Selkoe KA, D'Aloia CC, Crandall ED, Iacchei M, Liggins L, Puritz JB (2016). A decade of seascape genetics: contributions to basic and applied marine connectivity. Mar Ecol Prog Ser.

[6] Riginos C, Liggins L (2013). Seascape genetics: populations, individuals, and genes marooned and adrift. Geogr Compass.

[7] Roy D, Hurlbut TR, Ruzzante DE (2012). Biocomplexity in a demersal exploited fish, white hake (Urophycis tenuis): depth-related structure and inadequacy of current management approaches. Can J Fish Aquat Sci.

[8] Lal MM, Southgate PC, Jerry DR, Zenger KR (2016). Fishing for divergence in a sea of connectivity: the utility of ddRADseq genotyping in a marine invertebrate, the black-lip pearl oyster Pinctada margaritifera. Mar Genomics.

[9] Waples RS (1998). Separating the wheat from the chaff: patterns of genetic differentiation in high gene flow species. J Hered.

[10] Waples RS, Gaggiotti O (2006). What is a population? An empirical evaluation of some genetic methods for identifying the number of gene pools and their degree of connectivity. Mol Ecol.

[11] Lal MM, Southgate PC, Jerry DR, Bosserelle C, Zenger KR (2017). Swept away: ocean currents and seascape features influence genetic structure across the 18,000 km indo-Pacific distribution of a marine invertebrate, the black-lip pearl oyster Pinctada margaritifera. BMC Genomics.

[12] Limborg MT, Helyar SJ, de Bruyn M, Taylor MI, Nielsen EE, Ogden R (2012). Environmental selection on transcriptome-derived SNPs in a high gene flow marine fish, the Atlantic herring (Clupea harengus). Mol Ecol.

[13] Nosil P, Funk DJ, Ortiz-Barrientos D (2009). Divergent selection and heterogeneous genomic divergence. Mol Ecol.

[14] Andre C, Larsson LC, Laikre L, Bekkevold D, Brigham J, Carvalho GR (2011). Detecting population structure in a high gene-flow species, Atlantic herring (Clupea harengus): direct, simultaneous evaluation of neutral vs putatively selected loci. Heredity.

[15] Benestan L, Gosselin T, Perrier C, Sainte-Marie B, Rochette R, Bernatchez L (2015). RAD genotyping reveals fine-scale genetic structuring and provides powerful population assignment in a widely distributed marine species, the American lobster (Homarus americanus). Mol Ecol.

[16] Gaggiotti OE, Bekkevold D, Jorgensen HBH, Foll M, Carvalho GR, Andre C (2009). Disentangling the effects of evolutionary, demographic, and environmental factors influencing genetic structure of natural populations: Atlantic herring as a case study. Evolution.

[17] Garner BA, Hand BK, Amish SJ, Bernatchez L, Foster JT, Miller KM (2016). Genomics in conservation: case studies and bridging the gap between data and application. Trends Ecol Evol.

[18] Larson WA, Seeb LW, Everett MV, Waples RK, Templin WD, Seeb JE (2014). Genotyping by sequencing resolves shallow population structure to inform conservation of Chinook salmon ( Oncorhynchus tshawytscha). Evol Appl.

[19] Benestan L, Quinn BK, Maaroufi H, Laporte M, Clark FK, Greenwood SJ (2016). Seascape genomics provides evidence for thermal adaptation and current-mediated population structure in American lobster (Homarus americanus). Mol Ecol.

[20] Aykanat T, Lindqvist M, Pritchard VL, Primmer CR (2016). From population genomics to conservation and management: a workflow for targeted analysis of markers identified using genome-wide approaches in Atlantic salmon Salmo salar. J Fish Biol.

[21] Loman NJ, Misra RV, Dallman TJ, Constantinidou C, Gharbia SE, Wain J (2012). Performance comparison of benchtop high-throughput sequencing platforms. Nat Biotechnol.

[22] Rusk N (2009). Cheap third-generation sequencing. Nat Methods.

[23] Rhoads A, Au KF (2015). PacBio sequencing and its applications. Geno Proteo Bioinform.

[24] Campbell NR, Amish SJ, Pritchard VL, McKelvey KS, Young MK, Schwartz MK (2012). Development and evaluation of 200 novel SNP assays for population genetic studies of westslope cutthroat trout and genetic identification of related taxa. Mol Ecol Resour.

[25] Pujolar JM, Jacobsen MW, Als TD, Frydenberg J, Munch K, Jonsson B (2014). Genome-wide single-generation signatures of local selection in the panmictic European eel. Mol Ecol.

[26] Candy JR, Campbell NR, Grinnell MH, Beacham TD, Larson WA, Narum SR (2015). Population differentiation determined from putative neutral and divergent adaptive genetic markers in eulachon (Thaleichthys pacificus, Osmeridae), an anadromous Pacific smelt. Mol Ecol Resour.

[27] Saha A, Hauser L, Kent M, Planque B, Neat F, Kirubakaran TG (2015). Seascape genetics of saithe (Pollachius virens) across the North Atlantic using single nucleotide polymorphisms. ICES J Mar Sci.

[28] Phillips Bruce, Matsuda Hirokazi (2011). A Global Review of Spiny Lobster Aquaculture. Recent Advances and New Species in Aquaculture.

[29] Booth JD, Phillips BF (1994). Early-life history of spiny lobster. Crustaceana.

[30] Berry PF (1974). A revision of the Panulirus Homarus-Group of Spiny Lobsters (Decapoda, Palinuridae). Crustaceana.

[31] Delghandi M, Goddard S, Jerry DR, Dao HT, Al Hinai MSN, Al-Amry W, et al. Novel genomic microsatellite markers for genetic population and diversity studies of tropical scalloped spiny lobster (*Panulirus homarus*) and their potential application in related Panulirus species. Genet Mol Res. 2016;15(2):gmr7846. 10.4238/gmr.15027846. 10.4238/gmr.15027846 27173289

[32] Rogers PP, Barnard RM, Johnston MD (2010). Lobster aquaculture a commercial reality: a review. J Mar Biol Ass Ind.

[33] Fu CH, Fanning LP (2004). Spatial considerations in the management of Atlantic cod off Nova Scotia. Canada North Amer J Fish Man.

[34] Lavery SD, Farhadi A, Farahmand H, Chan TY, Azhdehakoshpour A, Thakur V (2014). Evolutionary divergence of geographic subspecies within the scalloped spiny lobster Panulirus homarus (Linnaeus 1758). PLoS One.

[35] Singh SP, Groeneveld JC, Al-Marzouqi A, Willows-Munro S (2017). A molecular phylogeny of the spiny lobster Panulirus homarus highlights a separately evolving lineage from the Southwest Indian Ocean. Peer J.

[36] Farhadi A, Jeffs AG, Farahmand H, Rejiniemon TS, Smith G, Lavery SD (2017). Mechanisms of peripheral phylogeographic divergence in the indo-Pacific: lessons from the spiny lobster Panulirus homarus. BMC Evol Biol.

[37] Al-Marzouqi A, Al-Amry W, Al-Hadabi A, Al-Senaidi R (2015). DNA barcoding of Panulirus homarus from Oman and Yemen. J Aquac Mar Biol..

[38] Reddy MM, Macdonald AHH, Groeneveld JC, Schleyer MH (2014). Phylogeography of the scalloped spiny-lobster Panulirus Homarus Rubellus in the Southwest Indian Ocean. J Crust Biol.

[39] Mehanna S, Al-Shijibi S, Al-Jafary J, Al-Senaidi R (2012). Population dynamics and management of scalloped spiny lobster Panulirus homarus in Oman coastal waters. J Biol Agr Heal.

[40] Book Fishery Statistics. Year Book Issued by Department of Fisheries Statistics. Ministry of Agriculture and Fisheries Wealth. Sultanate of Oman; 2016.

[41] Al-Marzouqi A, Al-Nahdi A, Jayabalan N, Groeneveld J (2007). An assessment of the spiny lobster Panulirus homarus fishery in Oman - another decline in the western Indian Ocean?. Western Indian Ocean J Mar Sci.

[42] Al-Marzouqi A, Chesalin M, Al-Shajibi S, Al-Hadabi A, Al SR (2015). Changes in the scalloped spiny lobster, Panulirus Homarus biological structure after a shift of the fishing season. J Aquac Mar Biol.

[43] Raymond M, Vaanto RL, Thomas F (1997). Rousset F, deMeeus T. Renaud F Heterozygote deficiency in the mussel species complex revisited Mar Ecol Prog Ser.

[44] Addison JA, Hart MW (2005). Spawning, copulation and inbreeding coefficients in marine invertebrates. Biol Lett.

[45] Zouros E (1990). Heterozygosity and growth in marine bivalves response. Evolution.

[46] Valles-Jimenez R, Cruz P, Perez-Enriquez R (2004). Population genetic structure of pacific white shrimp (Litopenaeus vannamei) from Mexico to Panama: microsatellite DNA variation. Mar Biotechnol.

[47] Brumfield RT, Beerli P, Nickerson DA, Edwards SV (2003). The utility of single nucleotide polymorphisms in inferences of population history. Trends Ecol Evol.

[48] Laurie CC, Doheny KF, Mirel DB, Pugh EW, Bierut LJ, Bhangale T (2010). Quality control and quality Assurance in Genotypic Data for genome-wide association studies. Genet Epidemiol.

[49] Li Heng (2014). Toward better understanding of artifacts in variant calling from high-coverage samples. Bioinformatics.

[50] Morse P, Kjeldsen SR, Meekan MG, McCormick MI, Finn JK, Huffard CL, et al. Genome-wide comparisons reveal a clinal species pattern within a holobenthic octopodthe Australian southern blue-ringed octopus, Hapalochlaena maculosa (Cephalopoda: Octopodidae). Ecol Evol. 2018;8(4):2253–67. 10.1002/ece3.3845 PMC5817145 29468041

[51] Nayfa MG, Zenger KR (2016). Unravelling the effects of gene flow and selection in highly connected populations of the silver-lip pearl oyster (Pinctada maxima). Mar Genomics.

[52] Stoffel MA, Esser M, Kardos M, Humble E, Nichols H, David P (2016). inbreedR: an R package for the analysis of inbreeding based on genetic markers. Methods Ecol Evol.

[53] Herdegen M, Dudka K, Radwan J (2014). Heterozygosity and orange coloration are associated in the guppy (Poecilia reticulata). J Evol Biol.

[54] Andrews KR, Luikart G (2014). Recent novel approaches for population genomics data analysis. Mol Ecol.

[55] Crooks L, Carlborg O, Marklund S, Johansson AM (2013). Identification of Null Alleles and Deletions from SNP Genotypes for an Intercross Between Domestic and Wild Chickens. G3-Gen Geno Genet.

[56] Kjeldsen SR, Zenger KR, Leigh K, Ellis W, Tobey J, Phalen D (2016). Genome-wide SNP loci reveal novel insights into koala (Phascolarctos cinereus) population variability across its range. Conserv Genet.

[57] Carlson CS, Smith JD, Stanaway IB, Rieder MJ, Nickerson DA (2006). Direct detection of null alleles in SNP genotyping data. Hum Mol Genet.

[58] Farhadi A, Farhamand H, Nematollahi MA, Jeffs A, Lavery SD (2013). Mitochondrial DNA population structure of the scalloped lobster Panulirus homarus (Linnaeus 1758) from the West Indian Ocean. ICES J Mar Sci.

[59] Yellapu B, Jeffs A, Battaglene S, Lavery SD (2017). Population subdivision in the tropical spiny lobster Panulirus ornatus throughout its indo-West Pacific distribution. ICES J Mar Sci.

[60] Dao Hoc Tan, Smith-Keune Carolyn, Wolanski Eric, Jones Clive M., Jerry Dean R. (2015). Oceanographic Currents and Local Ecological Knowledge Indicate, and Genetics Does Not Refute, a Contemporary Pattern of Larval Dispersal for The Ornate Spiny Lobster, Panulirus ornatus in the South-East Asian Archipelago. PLOS ONE.

[61] Iacchei M, O'Malley JM, Toonen RJ (2014). After the gold rush: population structure of spiny lobsters in Hawaii following a fishery closure and the implications for contemporary spatial management. Bull Mar Sci.

[62] Abdullah MF (2014). Alimuddin, Muththalib M, Salama AJ, Imai H. Genetic isolation among the northwestern, southwestern and central-eastern Indian Ocean populations of the pronghorn spiny lobster Panulirus penicillatus. Int J Mol Sci.

[63] Iacchei M, Gaither MR, Bowen BW, Toonen RJ (2016). Testing dispersal limits in the sea: range-wide phylogeography of the pronghorn spiny lobster Panulirus penicillatus. J Biogeogr.

[64] George RW (2005). Evolution of life cycles, including migration, in spiny lobsters (Palinuridae). N Z J Mar Freshw Res.

[65] Ivanochko, T. S. 2005. Sub-orbital scale variations in the intensity of the Arabian Sea monsoon. University of Edinburgh, PhD Thesis.

[66] Schott FA, McCreary JP (2001). The monsoon circulation of the Indian Ocean. Prog Oceano.

[67] Condie S, Condie R (2016). Retention of plankton within ocean eddies. Glob Ecol Biogeog.

[68] Chiswell SM, Roemmich D (1998). The east cape current and two eddies: a mechanism for larval retention?. N Z J Mar Freshw Res.

[69] Gonzalez EB, Knutsen H, Jorde PE (2016). Habitat discontinuities separate genetically divergent populations of a rocky shore marine fish. PLoS One.

[70] Simpson SD, Harrison HB, Claereboudt MR, Planes S (2014). Long-distance dispersal via ocean currents connects omani clownfish populations throughout entire species range. PLoS One.

[71] Johnson MS, Black R (1982). Chaotic genetic patchiness in an intertidal limpet. Siphonaria sp Mar Biol.

[72] Larson RJ, Julian RM (1999). Spatial and temporal genetic patchiness in marine populations and their implications for fisheries management. Calif Coop Ocean Fish Investig Reports.

[73] Nash WJ, Goddard M, Lucas JS (1988). Population genetic studies of the crown-of-thorns starfish, Acanthaster planci (L.), in the great barrier reef region. Coral Reefs.

[74] David P, Perdieu M (1997). A, Pernot a F, Jarne P. Fine-grained spatial and temporal population genetic structure in the marine bivalve Spisula ovalis. Evolution.

[75] Flowers JM, Schroeter SC, Burton RS (2002). The recruitment sweepstakes has many winners: genetic evidence from the sea urchin Strongylocentrotus purpuratus. Evolution.

[76] Selwyn JD, Hogan JD, Downey-Wall AM, Gurski LM, Portnoy DS, Heath DD (2016). Kin-aggregations explain chaotic genetic patchiness, a commonly observed genetic pattern, in a marine fish. PLoS One.

[77] Christie MR, Johnson DW, Stallings CD, Hixon MA (2010). Self-recruitment and sweepstakes reproduction amid extensive gene flow in a coral-reef fish. Mol Ecol.

[78] Iacchei M, Ben-Horin T, Selkoe KA, Bird CE, García-Rodríguez FJ, Toonen RJ (2013). Combined analyses of kinship and FSTsuggest potential drivers of chaotic genetic patchiness in high gene-flow populations. Mol Ecol.

[79] Zhao S, Zheng P, Dong S, Zhan X, Wu Q, Guo X (2013). Whole-genome sequencing of giant pandas provides insights into demographic history and local adaptation. Nat Genet.

[80] Dillon Shannon, McEvoy Rachel, Baldwin Darren S., Rees Gavin N., Parsons Yvonne, Southerton Simon (2014). Characterisation of Adaptive Genetic Diversity in Environmentally Contrasted Populations of Eucalyptus camaldulensis Dehnh. (River Red Gum). PLoS ONE.

[81] Zhan X, Dixon A, Batbayar N, Bragin E, Ayas Z, Deutschova L (2015). Exonic versus intronic SNPs: contrasting roles in revealing the population genetic differentiation of a widespread bird species. Heredity.

[82] Drury C, Dale KE, Panlilio JM, Miller SV, Lirman D, Larson EA (2016). Genomic variation among populations of threatened coral: Acropora cervicornis. BMC Genomics.

[83] Nielsen EE, Hemmer-Hansen J, Larsen PF, Bekkevold D (2009). Population genomics of marine fishes: identifying adaptive variation in space and time. Mol Ecol.

[84] Tsumura Y, Uchiyama K, Moriguchi Y, Ueno S, Ihara-Ujino T (2012). Genome scanning for detecting adaptive genes along environmental gradients in the Japanese conifer, Cryptomeria japonica. Heredity.

[85] Orsini L, Mergeay J, Vanoverbeke J, De Meester L (2013). The role of selection in driving landscape genomic structure of the waterflea Daphnia magna. Mol Ecol.

[86] Mohan R (1997). Size structure and reproductive variation of the spiny lobster Panulirus homarus over a relatively small geographic range along the Dhofar coast in the Sultanate of Oman. R Mar Freshw Res.

[87] Adamkewicz SL, Harasewych MG (1996). Systematics and biogeography of the genus Donax (Bivalvia: Donacidae) in eastern North America. Amer Malacol Bulletin.

[88] Jaccoud D, Peng K, Feinstein D, Kilian A (2001). Diversity arrays: a solid state technology for sequence information independent genotyping. Nuc Acid Res.

[89] Kilian A, Wenzl P, Huttner E, Carling J, Xia L, Blois H (2012). Diversity arrays technology: a generic genome profiling technology on open platforms. Methods Mol Biol.

[90] Sansaloni C, Petroli C, Jaccoud D, Carling J, Detering F, Grattapaglia D, Kilian A (2011). Diversity arrays technology (DArT) and next-generation sequencing combined: genome-wide, high throughput, highly informative genotyping for molecular breeding of Eucalyptus. BMC Proc.

[91] Lind C, Kilian A, Benzie J (2017). Development of diversity arrays technology markers as a tool for rapid genomic assessment in Nile tilapia. Anim Genet.

[92] Steinig E, Guppy J, Jones D, Zenger K. 2016. DartQC pipeline. https://github.com/esteinig/dartQC (2018). Accessed 5 Apr 2018.

[93] Li W, Godzik A (2006). Cd-hit: a fast program for clustering and comparing large sets of protein or nucleotide sequences. Bioinformatics.

[94] Purcell Shaun, Neale Benjamin, Todd-Brown Kathe, Thomas Lori, Ferreira Manuel A.R., Bender David, Maller Julian, Sklar Pamela, de Bakker Paul I.W., Daly Mark J., Sham Pak C. (2007). PLINK: A Tool Set for Whole-Genome Association and Population-Based Linkage Analyses. The American Journal of Human Genetics.

[95] Excoffier L, Laval G, Schneider S. Arlequin ( version 3 . 0 ): An integrated software package for population genetics data analysis. 2005;23(1):47–50. PMC2658868 19325852

[96] Belkhir K, Borsa P, Chikhi L, Raufaste N, Bonhomme F. 1996. GENETIX 4.05, logiciel sous Windows TM pour la génétique des populations. Universite´ de Montpellier II. http://www.genetix.univ-montp2.fr/. Accessed 5 Apr 2018.

[97] Do C., Waples R. S., Peel D., Macbeth G. M., Tillett B. J., Ovenden J. R. (2013). NeEstimatorv2: re-implementation of software for the estimation of contemporary effective population size (Ne) from genetic data. Molecular Ecology Resources.

[98] Weir B, Cockerham C (1984). Estimating F statistics for the analysis of population structure. Evolution.

[99] Excoffier L, Lischer HEL (2010). Arlequin suite ver 3.5. 5 a new Ser. Programs to perform Popul. Genet. Anal. Under {Linux} {windows}. {molecular} Ecol. Resour.

[100] Sheppard CRC, Salm RV (1988). Reef and coral communities of Oman, with a description of a new coral species (order scleractinia, genus acanthastrea). J Nat Hist.

[101] Schils T, Wilson SC (2006). Temperature threshold as a biogeographic barrier in northern Indian ocean macroalgae. J Phycol.

[102] Jombart T (2008). Adegenet: a R package for the multivariate analysis of genetic markers. Bioinformatics.

[103] Jombart T, Devillard S, Balloux F (2010). Discriminant analysis of principal components: a new method for the analysis of genetically structured populations. BMC Genet.

[104] Steinig EJ, Neuditschko M, Khatkar MS, Raadsma HW, Zenger KR (2016). NETVIEW P: a network visualization tool to unravel complex population structure using genome-wide SNPs. Mol Ecol Resour.

[105] Neuditschko M, Khatkar MS, Raadsma HW (2012). NETVIEW: a high-definition network-visualization approach to detect fine-scale population structures from genome-wide patterns of variation. PLoS One.

[106] Tamura K, Stecher G, Peterson D, Filipski A, Kumar S (2013). MEGA6: molecular evolutionary genetics analysis version 6.0. Mol Biol Evol.

[107] Foll M, Gaggiotti O (2008). A genome-scan method to identify selected loci appropriate for both dominant and Codominant markers: a Bayesian perspective. Genetics.

[108] Beaumont MA, Nichols RA (1996). Evaluating loci for use in the genetic analysis of population structure. Proc R Soc B Biol Sci.

[109] Beaumont MA, Balding DJ (2004). Identifying adaptive genetic divergence among populations from genome scans. Mol Ecol.

[110] Foll M. BayeScan v2.1 user manual. Ecology. 2012;20:1450–62.

