# Supplementary material for: Genome-wide SNP analyses reveal high gene flow and signatures of local adaptation among the scalloped spiny lobster (Panulirus homarus) along the Omani coastline
Source: BMC Genomics. 2018 Sep 19;19:690. doi: 10.1186/s12864-018-5044-8 (PMC6146514; doi:10.1186/s12864-018-5044-8)
Supplement: Supplementary file 3 — Genotypic data of P. homarus for 164 individuals from Oman using 504 putatively directional SNPs. Genotypes are in genetix format. (PDF 389 kb) [file 12864_2018_5044_MOESM3_ESM.pdf]

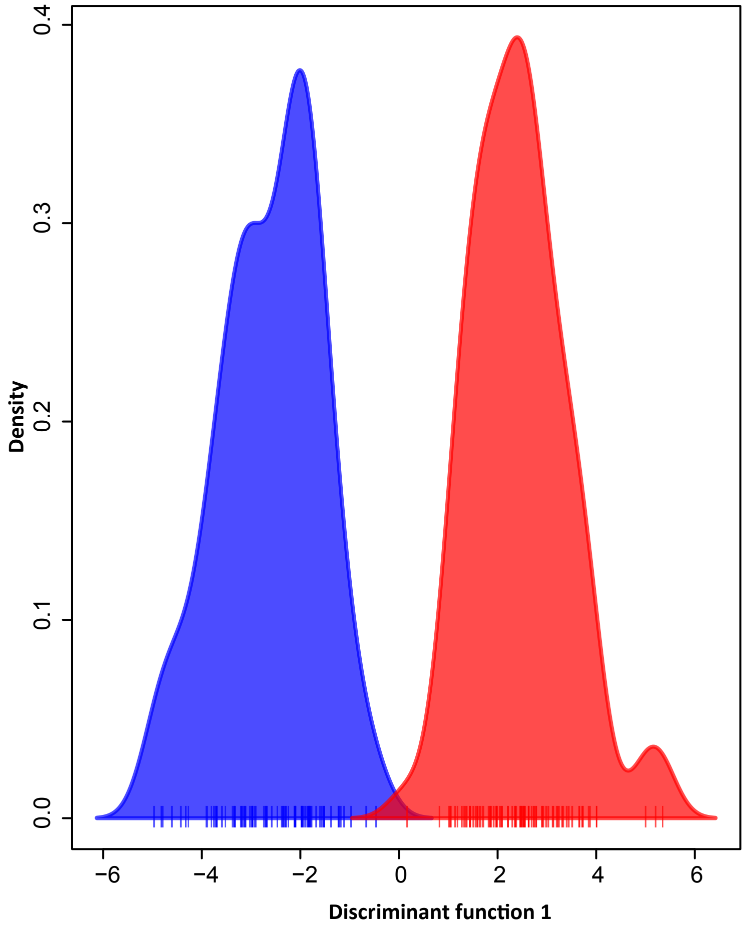

Additional file 2 A plot of the Discriminant Analysis of Principal Components (DAPC) against the discriminant function retained, indicating presence of two genetic clusters of *P. homarus* in Oman. The plot was generated using the most informative 13 PCs identified from all 7,988 SNPs dataset across 164 *P. homarus* individuals in the R package *adegenet*.
